# Homebased screening for cognitive impairment due to dementia

**DOI:** 10.1192/j.eurpsy.2022.1677

**Published:** 2022-09-01

**Authors:** T. Hansen, F. Waldorff, K. Andersen, E. Stenager

**Affiliations:** 1 The Region of Southern Denmark, Research Unit, Mental Health, Children And Adult, Aabenraa, Aabenraa, Denmark; 2 University of Copenhagen, Department Of Public Health, The Research Unit For General Practice And Section Of General Practice, København K, Denmark; 3 Region of Southern Denmark, Department Of Mental Health Odense, Odense, Denmark

**Keywords:** screening, Dementia, cognitive impairment

## Abstract

**Introduction:**

Dementia develops slowly and insidiously and causes cognitive impairment. The diagnosis is pivotal for relevant treatment and care. However, 50,000 people are estimated to have undiagnosed dementia in Denmark, while 36,000 are diagnosed.

The municipalities offers a home visit to the population at the ages of 75 and 80 years to assess the need of care and prevent sickness. These home visits are well established and might offer an unused opportunity to detect cognitive impairment and dementia.

**Objectives:**

To assess impaired cognition at home visits in order to initiate clinical examination for dementia.

**Methods:**

A feasibility study with the use of Brief Assessment of Impaired Cognition Questionnaire (BASIC-Q) (sensitivity 0.92, specificity 0.97) at home visits. It is expected to include 1000 participants without a dementia diagnosis at the ages of 75 and 80 years. Participants will be included in a period of 12 moths (in the year of 2022), in a number of municipalities.

If the screening for cognitive impairment is positive, the participant is motivated for clinical examination at the general practitioner. Follow-up through registers and general practitioners.

**Results:**

Preliminary results will be presented at the conference.

**Conclusions:**

Assessment of cognition might give an opportunity to start medication and social support early in the elderly with impaired cognition and undiagnosed dementia.

**Disclosure:**

No significant relationships.

